# The property of the Japanese version of the Recovery Knowledge Inventory (RKI) among mental health service providers: a cross sectional study

**DOI:** 10.1186/s13033-017-0178-7

**Published:** 2017-12-28

**Authors:** Rie Chiba, Maki Umeda, Kyohei Goto, Yuki Miyamoto, Sosei Yamaguchi, Norito Kawakami

**Affiliations:** 1 Research Institute of Nursing Care for People and Community, University of Hyogo, 13-71 Kitaoji-cho, Akashi, Hyogo 673-8588 Japan; 2 Department of Public Health Nursing, Graduate School of Nursing, St Luke’s International University, 10-1, Akashi-cho, Chuo-ku, Tokyo, 104-0044 Japan; 3 Tokyo Musashino Hospital, 4-11-11, Komone, Itabashi-ku, Tokyo, 173-0037 Japan; 4 Department of Psychiatric Nursing, Graduate School of Medicine, The University of Tokyo, 7-3-1, Hongo, Bunkyo-ku, Tokyo, 113-0033 Japan; 5 Department of Psychiatric Rehabilitation, Institute of Mental Health, National Center of Neurology and Psychiatry, 4-1-1, Ogawahigashi-cho, Kodaira-shi, Tokyo, 187-8553 Japan; 6 Department of Mental Health, Graduate School of Medicine, The University of Tokyo, 7-3-1, Hongo, Bunkyo-ku, Tokyo, 113-0033 Japan

**Keywords:** Cross-sectional survey, Japan, Knowledge, Mental health services, Professional, Recovery, Reliability, Scale, Validity

## Abstract

**Background:**

The Recovery Knowledge Inventory (RKI) is one of the influential scales to assess knowledge and attitude toward recovery-oriented practices among mental health service providers. In the present study, we aimed to develop a Japanese version of RKI and examine the validity and reliability.

**Methods:**

We translated RKI into Japanese by reference to the guidelines for translating and adapting psychometric scales. A cross-sectional questionnaire survey was conducted with mental health service providers. Of a total of 475 eligible professionals, we used data from the 299 participants without missing value for the analyses (valid response rate = 62.9%). The questionnaire included Japanese RKI, Recovery Attitudes Questionnaire, The positive attitudes scale, and Japanese-language version of the Social Distance Scale. To examine the factorial validity of RKI, explanatory factor analysis and confirmatory factor analysis was employed. Convergent validity was assessed by calculating Pearson’s correlation coefficients between the total RKI score and the scores for the other three scales. We also calculated Cronbach’s α coefficients for the total score and for each domain of RKI to assess internal consistency reliability.

**Results:**

The participants’ mean age was 40.4 years and 30.4% were men. 20-item RKI did not provide any adequate or interpretable factor solutions at any number of factors by EFAs. Thus four items (#1, 4, 5, and 13) were subsequently eliminated in stages, then 16-item RKI was employed as a consequence for further analyses. EFA with four factor structures yielded marginally interpretable constitution. Each factor represented the knowledge regarding psychiatric symptoms and recovery; knowledge about the recovery process; the understanding of what is important for recovery; and the understanding of the challenges and responsibility in recovery, respectively. Subsequent CFA suggested good fit to the data. Good convergent validity and understandable internal consistency reliability were also observed.

**Conclusions:**

The Japanese 16-item RKI revealed reasonable factorial validity, good convergent validity, and understandable internal consistency reliability among mental health professionals. Japanese cultural settings seemed to influence the four-factor structure in the present study. It can be used for future study in Japan, while future large-scale research is required to ensure robust verification.

## Background

“Recovery”, also known as “personal recovery”, in the context of mental health is a unique personal process of transformation. It includes subjective discovery of a new self to overcome mental illness and regain control and responsibility of one’s own life [1, 2]. The recovery paradigm has been an international policy which campaigns for better mental healthcare [3]. However, a few earlier studies have argued that attitudes among mental health service providers towards recovery are more pessimistic, prejudiced, or qualitatively different compared to that among people with mental illness [4–6]. Since such negative attitude towards recovery among mental health service providers could hinder recovery-focused healthcare, some researchers have indicated the need for, and difficulty in recovery orientated mental health services [7–10].

To date, various scales pertaining to recovery orientation have been developed, including individual’s attitude toward recovery for use with mental health service providers or people with mental illness [11–13], competence to promote recovery [14, 15], and the recovery orientation of services [16]. Among such scales, the Recovery Knowledge Inventory (RKI), developed in the USA, is one of the influential and predominantly used scales to assess knowledge and attitude towards recovery-oriented practices among mental health service providers [17]. RKI has revealed some factors associated with recovery orientation in cross-sectional studies [18–20], as well as in longitudinal studies [21]. Appreciably, in recent years, RKI has been notably used in several interventional studies in Australia [22–25], USA [26], Canada [27, 28], UK [29], and Netherlands [30], as various educational programs have been developed for service providers to enhance recovery orientation.

The preliminary version of RKI comprised 36 items, which was reduced to 20 items based on the results of principal component analysis and feedback from stakeholders for refinement [17]. Then Bedregal et al. [17] empirically demonstrated a four-factor structure for the 20-item RKI: (1) roles and responsibilities in recovery, (2) non-linearity of the recovery process, (3) the roles of self-definition and peers in recovery, and (4) expectations regarding recovery. Cronbach’s α coefficients for each domain were .81, .70, .63, and .47, respectively [17].

Although several earlier studies using RKI employed the 20-item scale with the four-factor structure, studies among nursing students in Australia [9] and the Dutch version of RKI among professionals [31] did not yield an acceptable fit for the structure. Thus, they suggested further elimination of certain RKI items. Considering the results of the analyses, Happell et al. [9] reduced the items to 16, whereas Wilrycx et al. [31] reduced them to 14, respectively. In addition, most of the earlier studies using RKI were conducted in Western countries, with the exception of a report in Hong Kong [32]. Thus, it is yet to be elucidated whether or not RKI is a useful tool in Asian countries. Considering that the concept of “recovery” differs among countries or cultures [33], further evaluation is clearly required to examine the cross-cultural applicability of RKI, including the context of Asian culture.

In the present study, we aimed to develop a Japanese version of RKI and examine the factorial validity, convergent validity, and internal consistency reliability of the Japanese version of RKI among mental health service providers in community and inpatient settings in Japan. We hypothesized that the Japanese version of RKI had good factorial validity, convergent validity, and internal consistency. The use of RKI would promote the understanding of recovery, and thus, encourage recovery-oriented care in Japan where traditional psychiatric treatment and paternalistic care through long-term inpatient treatment has been prevalent for a long period.

## Methods

### Participants

A cross-sectional questionnaire survey was implemented with mental health service providers from February to March 2012. Eligible professions included psychiatrists, registered or assistant nurses, public health nurses, clinical psychologists, pharmacists, occupational therapists, and social workers. Participants came from two psychiatric hospitals in the Kanto region, as well as a total of 56 psychiatric clinics and community service agencies in Tokyo, Japan.

In the two psychiatric hospitals, there were 220 eligible professionals, of whom 180 agreed to participate and returned completed questionnaires. In the psychiatric clinics and community service agencies, there were 255 eligible professionals, of whom 151 agreed to participate and responded to the questionnaire. This gave a total of 331 respondents; however, 32 were excluded because of missing responses for one or more of the items on RKI. We used data from the remaining 299 participants for the analyses (valid response rate = 62.9%).

### Measures

#### Development of the Japanese version of RKI

On the RKI, higher total scores mean greater knowledge and more positive attitudes towards the concept of recovery [17]. All items follow a Likert-style response format ranging from 1 (strongly disagree) to 5 (strongly agree). Of the total 20 items, 15 are scored inversely to minimize the effect of social desirability. Items are shown in Table 2.

We translated the RKI into Japanese with the consent of the original developer, by reference to the guidelines for translating and adapting psychometric scales [34, 35]. We developed the Japanese RKI in accordance with the following five procedures. (1) Forward translation: two of the authors separately translated RKI from English into Japanese. (2) Reconciliation: five of the authors went through them and gained consensus on a draft Japanese translation of RKI that best reflected the literal and conceptual content of the original English version. (3) Cognitive debriefing and review of the cognitive debriefing results: after we reworded some items as needed, two mental health service providers, a peer-support group leader with chronic mental illness, and five peer-support group members tested the Japanese RKI. (4) Back-translation: two native English-speaking professional translators, who did not know the original RKI, back-translated the Japanese version into English. (5) Back-translation review and finalization: all of us reviewed the back translations by comparison with the original RKI, and assured the literal and conceptual equivalence between the original and Japanese RKIs.

#### Recovery Attitudes Questionnaire (RAQ)

The seven-item RAQ is a five-point Likert scale to assess attitudes toward, and knowledge of, recovery [11]. Items such as “To recover requires faith” or “People in recovery sometimes have setbacks” constitute two domains, i.e., “recovery is possible and needs faith” and “recovery is difficult and differs among people”. Higher total scores indicate a more positive attitude to the concept of recovery. A study in USA has revealed the reasonable internal consistency reliability (Cronbach’s α = .70), marginally acceptable test–retest reliability (*r* = .67), and reasonable factorial validity of RAQ [11]. The Japanese version of RAQ revealed acceptable validity (GFI = .95; AGFI = .90; CFI = .86) and mediocre reliability (Cronbach’s α = .64; ICC for test–retest reliability = .68) [36]. RAQ was assumed to be positively associated with RKI.

#### The positive attitudes scale

The positive attitudes scale is a scale to assess one’s positive attitudes toward people with mental illness. Nineteen items including “I think most people with mental illness have ability to understand their illness” comprise three domains such as “expectations for the abilities and recovery”, “attitudes toward living alongside people with mental illness”, and “supportive helping behaviors”. Items require responses on a four-point Likert scale ranging from “strongly disagree” to “strongly agree”, with a higher scores indicating a more positive attitude toward people with mental illness. Good internal consistency reliability and convergent validity have been confirmed for the positive attitudes scale among mental health service providers in Japan (Cronbach’s α = .88) [37]. The positive attitudes scale was supposed to be positively associated with RKI.

#### Japanese-language version of the Social Distance Scale (SDSJ)

The SDSJ is a scale to assess people’s desire and perception for social distance toward people with schizophrenia and it is not limited to professionals. It includes items such as “I do not want to take a taxi with a driver who has a history of schizophrenia”, which require responses on a four-point Likert scale ranging from “disagree” to “agree”, with a higher scores indicating a more negative attitude. The SDSJ has good internal consistency reliability (Cronbach’s α = .70), good test–retest reliability (*r* = .95), and acceptable factorial validity (GFI = .94; AGFI = .81; CFI = .90) [38]. SDSJ was assumed to be negatively associated with RKI.

### Statistical analysis

The suitability of the data for factor analysis was first examined using the Kaiser–Mayer–Olkin (KMO) measure of sampling adequacy and Bartlett’s Chi square test of sphericity. Subsequently, the KMO indicator was assessed by the adequacy criteria (meritorious > .80) [39]. To extract a factor structure of the scale items for the assessment of factor-based validity, we first applied an explanatory factor analysis (EFA) using the maximum likelihood method and promax rotation, from one- to four-factor structures with reference to earlier studies [9, 17, 31]. In the case that these analyses failed to provide any admissible solution, we implemented further EFA by step-by-step elimination of some items to refine the factor structure with reference to earlier studies [9, 17, 31].

Then a confirmatory factor analysis (CFA) was employed to test the fitness of the data to the factor structure extracted by EFA in this study. We also conducted CFAs for the data of the original four-factor structure [17], as well as for the three-factor structure of the 16-item version [9] and the one-factor structure of the 14-item version [31] for reference. Model fit was assessed using a combination of fit indices, including the goodness-of-fit (GFI), the adjusted goodness-of-fit (AGFI), the comparative fit index (CFI), and the root mean square error of approximation (RMSEA). The acceptability of model fit was judged by the recommended standards of GFI, AGFI, and CFI greater than .90, and RMSEA values of .06 or less, for a close fit [40]. Convergent validity was assessed by calculating Pearson’s correlation coefficients between the total RKI score and the scores for the other three scales, i.e., RAQ, positive attitudes scale, and SDSJ.

We calculated Cronbach’s α coefficients for the total score and for each domain of the RKI to assess internal consistency reliability. Cronbach’s α of no less than .70　was assumed to be acceptable [31].

All statistical analyses including descriptive analyses, but excluding CFA, were conducted using IBM SPSS Statistics Base version 24.0. CFA was conducted in IBM SPSS Amos version 24.0 using structural equation modeling. *p* values of less than .05 were considered statistically significant (two-tailed tests).

## Results

### Participant characteristics

The participants’ sociodemographic and occupational characteristics are shown in Table 1. Most of the participants were female (about 70%), and the mean age was 40.4 years (range: 22–75 years). The mean length of experience in psychiatric services was 9.9 years (range: 0–45 years). The largest professional group was registered or assistant nurses. In this study, all doctors were psychiatrists because of the absence of general practitioner/family physician under the Japanese health care system. Regarding the educational background, most health service providers had a bachelor’s degree or higher, whereas some associate nurses were high school graduates.

**Table 1:** Participants’ socio-demographic and occupational characteristics (*N* = 299)

Variables	*n* [Mean]	(%) [SD]
Sex		
Female	206	(68.9)
Male	91	(30.4)
Unknown	2	(0.7)
Age (years)	[40.4]	[11.9]
Years of work tenure in psychiatric or mental health services	[9.9]	[8.5]
Occupation		
Registered nurse/assistant nurse	132	(44.1)
Social worker	103	(34.4)
Occupational therapist	20	(6.7)
Clinical psychologist	20	(6.7)
Psychiatrist	14	(4.7)
Pharmacist	5	(1.7)
Public health nurse	3	(1.0)
Others	2	(0.7)
Department		
Ward	129	(43.1)
Job assistance	60	(20.1)
Out-patient clinic/Home-visit nursing	40	(13.4)
Psychiatric day-care	26	(8.7)
Home assistance/rehabilitation	15	(5.0)
Group home	5	(1.7)
Community activity support center	2	(0.7)
Others, Unknown	22	(7.3)
Employment status		
Full-time job	238	(79.6)
Part-time job	53	(17.7)
Unknown	8	(2.7)
Education		
High school	6	(2.0)
Vocational school	117	(39.2)
Junior college	18	(6.0)
College	122	(40.8)
Graduate school	33	(11.0)
Unknown	3	(1.0)

### Validity of RKI

#### Factorial validity

The mean total 20-item RKI score was 66.1 [standard deviation (SD) = 6.6; range: 48–89]. There was no item with either a ceiling or floor effect (Table 2).

**Table 2:** Descriptive statistics for the 20-item RKI (*N* = 299)

No.	Items	Mean	*SD*	Min	Max
1	The concept of recovery is equally relevant to all phases of treatment	2.93	.95	1	5
2	People receiving psychiatric/substance abuse treatment are unlikely to be able to decide their own treatment and rehabilitation goals	3.98	.77	2	5
3	All professionals should encourage clients to take risks in the pursuit of recovery	2.74	.84	1	5
4	Symptom management is the first step towards recovery from mental illness/substance abuse	2.31	.79	1	4
5	Not everyone is capable of actively participating in the recovery process	2.44	.88	1	5
6	People with mental illness/substance abuse should not be burdened with the responsibilities of everyday life	3.86	.73	2	5
7	Recovery in serious mental illness/substance abuse is achieved by following a prescribed set of procedures	3.55	.77	1	5
8	The pursuit of hobbies and leisure activities is important for recovery	4.16	.57	3	5
9	It is the responsibility of professionals to protect their clients against possible failures and disappointments	3.36	.95	1	5
10	Only people who are clinically stable should be involved in making decisions about their care	3.91	.78	2	5
11	Recovery is not as relevant for those who are actively psychotic or abusing substances	3.61	.90	1	5
12	Defining who one is, apart from his/her illness/condition, is an essential component of recovery	3.90	.75	1	5
13	It is often harmful to have too high of expectations for clients	2.32	.77	1	5
14	There is little that professionals can do to help a person recover if he/she is not ready to accept his/her illness/condition or need for treatment	3.67	.76	1	5
15	Recovery is characterized by a person making gradual steps forward without major steps back	3.49	.85	1	5
16	Symptom reduction is an essential component of recovery	2.65	.78	1	5
17	Expectations and hope for recovery should be adjusted according to the severity of a person’s illness/condition	2.47	.83	1	5
18	The idea of recovery is most relevant for those people who have completed, or are close to completing, active treatment	3.40	.77	1	5
19	The more a person complies with treatment, the more likely he/she is to recover	3.40	.77	2	5
20	Other people who have a serious mental illness or are recovering from substance abuse can be as instrumental to a person’s recovery as mental health professionals	3.96	.68	2	5

The following fifteen item scores were reversed before computing the statistics: No. 2, 4, 5, 6, 7, 9, 10, 11, 13, 14, 15, 16, 17, 18, and 19

The 20-item RKI did not provide any adequate or interpretable factor solutions at any number of factors by EFAs. Thus, four items (#1, 4, 5, and 13) were subsequently eliminated in stages, and consequently, the 16-item RKI was employed for further analyses.

The KMO was .84, indicating meritorious sampling adequacy. Bartlett’s test of sphericity was statistically significant (approximate Chi square = 1074, *df* = 120, *p* < .001), suggesting that there were correlations between the variables. EFA with four-factor structures yielded a marginally interpretable constitution (Table 3). In the present study, Factor 1 represents knowledge regarding psychiatric symptoms and recovery, Factor 2 denotes knowledge about the recovery process, Factor 3 indicates the understanding of what is important for recovery, and Factor 4 denotes the understanding of the challenges and responsibility in recovery. Inconsistent with the theoretical assumption, item #3 “All professionals should encourage clients to take risks in the pursuit of recovery” was negatively loaded on Factor 4 with a factor loading of .32.

**Table 3:** Factors derived from the Japanese version of the 16-item RKI: an item factor analysis with the maximum likelihood method and promax rotation (*N* = 299)

No.	Items	Factor			
		1	2	3	4
11	Recovery is not as relevant for those who are actively psychotic or abusing substances	.82			
10	Only people who are clinically stable should be involved in making decisions about their care	.74			
2	People receiving psychiatric/substance abuse treatment are unlikely to be able to decide their own treatment and rehabilitation goals	.45			
14	There is little that professionals can do to help a person recover if he/she is not ready to accept his/her illness/condition or need for treatment	.44			
19	The more a person complies with treatment, the more likely he/she is to recover		.73		
18	The idea of recovery is most relevant for those people who have completed, or are close to completing, active treatment		.64		
16	Symptom reduction is an essential component of recovery		.45		
17	Expectations and hope for recovery should be adjusted according to the severity of a person’s illness/condition		.41		
15	Recovery is characterized by a person making gradual steps forward without major steps back		.32		
12	Defining who one is, apart from his/her illness/condition, is an essential component of recovery			.59	
20	Other people who have a serious mental illness or are recovering from substance abuse can be as instrumental to a person’s recovery as mental health professionals			.58	
8	The pursuit of hobbies and leisure activities is important for recovery			.56	
6	People with mental illness/substance abuse should not be burdened with the responsibilities of everyday life				.54
7	Recovery in serious mental illness/substance abuse is achieved by following a prescribed set of procedures				.54
9	It is the responsibility of professionals to protect their clients against possible failures and disappointments				.39
3	All professionals should encourage clients to take risks in the pursuit of recovery				− .32

The following twelve item scores were reversed before computing the statistics: No. 2, 6, 7, 9, 10, 11, 14, 15, 16, 17, 18, and 19

Subsequent CFA suggested good fit to the data; GFI, AGFI, CFI, and RMSEA reached the recommended standards (GFI = .93; AGFI = .90; CFI = .91; RMSEA = .053) (Table 4). This factor structure resulted in the best fit to the data over the three-factor 16-item version [9] and one-factor 14-item version of RKI [31]. The four-factor structure in the original study [17] resulted in an unfitting solution in this sample (Table 4).

**Table 4:** Results of confirmatory factor analysis: comparison of goodness-of-fit indices between four-, three-, and one-factor RKI models (*N* = 299)

Model	GFI	AGFI	CFI	RMSEA	Chi square	*df*	*p*
*4-factor* ^*a*^	*.93*	*.90*	*.91*	*.053*	*179.59*	*98*	*.00*
3-factor^b^	.89	.85	.76	.080	295.63	101	.00
1-factor^c^	.87	.82	.77	.093	274.96	77	.00

Four-factor model derived in the original study by Bedregal et al. [17] is not shown because of an improper solution in this study

*GFI* goodness of fit index, *AGFI* adjusted goodness of fit index; *CFI* confirmatory fit index, *df* degrees of freedom; better fit model denoted by italic letters

^a^20 items loaded on a four-factor structure in the present study

^b^16 items loaded on a three-factor structure by Happell et al. [9]

^c^14 items loaded on one factor structure by Wilrycx et al. [31]

#### Convergent validity

The mean total score of the 16-item RKI was significantly and positively correlated with both the mean RAQ total score (*r* = .34; *p* < .01) and the mean total score for the positive attitudes scale (*r* = .46; *p* < .01). Conversely, the mean total score of the 16-item RKI score was significantly and negatively correlated with the mean total SDSJ score (*r* = − .46; *p* < .01).

### Reliability of RKI

Cronbach’s α coefficient was .77 for the total 16-item RKI score, with Factor 1 for .75, Factor 2 for.66, Factor 3 for .59, and Factor 4 for .24.

## Discussion

In the current study, we developed the Japanese version of the RKI. After omitting four items (#1, 4, 5, and 13), the resulting Japanese 16-item RKI revealed reasonable factorial validity, good convergent validity, and poor to good internal consistency reliability among mental health professionals.

We considered the elimination of these four items to be adequate because item #1 was also omitted in both of the earlier studies [9, 31]. Items #4, 5, and 13 had the lowest mean scores; in addition, items #5 and 13 had insufficient factorial attribution in the earlier study [17].

Explanatory factor analysis revealed a four-factor structure different from the one in the original study [17], even though they also had structural similarity. Factor 1 in the present study, representing knowledge regarding psychiatric symptoms and recovery, had three items in common with Factor 1 in the original study, whereas Factor 2 in the present study, representing knowledge about the process of recovery, had four items in common with Factor 2 in the original study. All three items in Factor 3 in the present study, representing the understanding of what is important for recovery, were included in Factor 3 in the original study. In contrast, the three items out of Factor 4 in the present study, about the understanding of the challenges and responsibility in recovery, emanated from Factor 1 in the original study. Although it is inconsistent compared to the earlier one [17], the current factor structure may provide a modest interpretation, given that successive CFA resulted in good levels of fit to the data. Nevertheless, the four-factor structure in the original study [17] did not yield a proper solution by the CFA, as in the earlier studies [9, 31].

Interestingly, item #3 “All professionals should encourage clients to take risks in the pursuit of recovery” negatively loaded on Factor 4 with a low factor loading. It might be affected by the therapeutic culture of the participants. In this study, > 40% of the participants came from ward settings; thus, our participants might be more protective and gave preference to safety over taking risks. It also seems to indicate a prevailing attitude that emphasizes the safe ground, reflecting the long history of prolonged hospitalizations for psychiatric treatment in Japan. According to Nonaka and Hirasawa [41], interdependence originating in collaborative agriculture culture is the foundation of traditional social structures in Japan. Thus, the harmonious attitude towards one’s life, rather than risk-taking behavior, is valued in Japan. Therefore, one can argue that this negative loading is construable in the context of Japanese culture. In addition, Factors 1 and 4 in the present study, stemming mainly from one factor in the original study [17], can also be explained by such specific settings in Japan. It means that, in Japan, knowledge regarding psychiatric symptoms and recovery is somehow independent of the understanding of challenges and responsibility in recovery (Appendix).

The 16-item RKI was significantly and positively correlated with RAQ and positive attitude scores, whereas the RKI score was significantly and negatively correlated with the SDSJ score. Although these correlations were weak, they were consistent with our hypotheses. The comparatively weak correlation between the RKI and RAQ scores suggests that they do not share the same assessment aspects, even though both the scales suitably assess knowledge and attitude towards recovery. The relationship observed in the present study was consistent with that in an earlier study in the Netherlands, which revealed a relatively weak positive relationship between the RKI and RAQ scores (*r* = .20; *p* = .004) [31]. The moderate correlation between RKI and positive attitude score suggests that these scales are related, although they are conceptually dissimilar. The positive attitude scale domains of “expectations for abilities and recovery” and “supportive helping behaviors” are similar to several RKI items. In contrast, the positive attitude domain of “attitudes toward living alongside people with mental illness” was not in accordance with the RKI items. Besides, the negative correlation between the RKI and SDSJ scores in the present study also demonstrated reasonable validity because previous conceptual and empirical studies have suggested that the recovery of people with mental illness can be impeded by negative attitude towards them [42–44].

The Cronbach’s α coefficient for the total 16-item RKI in the present study indicated good internal consistency reliability. This finding was consistent with the results of studies undertaken by service providers in Australia (α = .78 [45], α = .83 [23], and α = .79 [24]). Alternatively, the Cronbach’s α for each domain indicated varying reliability from good to poor. This fairly broad result was also similar to those in the earlier studies (α = .47–.81 [17], α = .49–.75 [9], α = .43–.79 [23], α = .45–.77 [45]). In particular, poor reliability indicated in Factor 4 in the present study was because of item #3 with negative factor loading. With respect to the scales to assess one’s knowledge, high internal consistency would not necessarily be expected, because people may possess knowledge in a certain area but may lack knowledge in others [46]. Thus, the lower reliability observed in the present study seems fathomable, given that RKI is a scale to assess one’s knowledge.

Considering that the current study supported a certain level of validity and reliability of the total 16-item RKI, the Japanese 16-item RKI can be used in future studies. However, Wilrycx et al. [31] reported that the composition and formulation of the RKI items were complex and difficult to interpret. Thus, the original RKI itself may be considered for the establishment of a more robust factor structure. In addition, a more careful review of Japanese wording and expression may improve the conceptual equivalence and conceptualize “recovery knowledge and recovery attitudes” in the context of the original RKI more concretely.

The “recovery” concept developed in Western countries is not the same between the Western, Asian countries [47–49], and even in the English-speaking countries [50]. Therefore, culturally different features of recovery attitude and knowledge must be the focus of future studies. Future cross-cultural studies may reveal internal and external aspects of influence on the knowledge and attitude towards recovery.

The present study has some limitations. First, test–retest reliability was not assessed. Second, three-quarters of the total study population were registered nurses, assistant nurses, or psychiatric social workers. Therefore, the generalizability of our findings may be limited to these occupations. Further large-scale research in a more diverse population of mental health service providers is required for robust verification of the Japanese version of RKI.

## Conclusions

The present study examined factorial validity, convergent validity, and internal consistency reliability of the Japanese version of RKI among mental health professionals. The Japanese 16-item RKI revealed reasonable factorial validity, good convergent validity, and understandable internal consistency reliability among mental health professionals. Japanese cultural settings seemed to influence the four-factor structure in the present study. Although the scale can be used for future study in Japan, future large-scale research is required to ensure robust verification.

## Appendix

See Table 5

**Table 5:** The Japanese version of the Recovery Knowledge Inventory (RKI)

No		まったく そう 思わない	そう 思わない	どちら とも いえない	そう思う	とても そう思う
1^a^	リカバリーの考え方は、治療のどの段階でも同じように適用できる	1	2	3	4	5
2^b^	**精神科治療やアルコール·薬物乱用の治療を受けている人が、自分の治療やリハビリテーションの目標を決めることはできないだろう**	1	2	3	4	5
3	どの専門職者も、相談者(利用者)に、リカバリーの追求のためなら思い切ってやってみるよう励ますべきである	1	2	3	4	5
4^a^	症状のコントロールは、精神の病気やアルコール·薬物乱用からのリカバリーへの第一歩である	1	2	3	4	5
5^a^	**リカバリーに積極的に取り組む力を誰もがもっているとは限らない**	1	2	3	4	5
6^b^	**精神の病気やアルコール・薬物乱用のある人は、日々の生活に生じるさまざまな責任を負うべきではない**	1	2	3	4	5
7^b^	**重い精神の病気やアルコール·薬物乱用におけるリカバリーは、決められた手順に沿えば実現する**	1	2	3	4	5
8	**趣味や余暇の活動を楽しむことは、リカバリーのために大切である**	1	2	3	4	5
9^b^	**相談者(利用者)が失敗や落胆をしないように守るのは、専門職者の責任である**	1	2	3	4	5
10^b^	**病状が安定している人だけが、自分のケアについて決めることに参加できる**	1	2	3	4	5
11^b^	**精神症状が激しい人やアルコール・薬物を乱用中の人には、リカバリーはあてはまらない**	1	2	3	4	5
12	**病気や状態にかかわらず、自分がどんな人間なのかを理解することは、リカバリーに不可欠な要素である**	1	2	3	4	5
13^a^	相談者(利用者)に対する高すぎる期待は、しばしば問題を引き起こす	1	2	3	4	5
14^b^	**自分の病気や状態、治療の必要性を受け入れる準備ができていない人には、リカバリーするために専門職者が手助けできることはほとんどない**	1	2	3	4	5
15^b^	**リカバリーには、大きな後戻りはせずに、徐々に前に進んでいくという特徴がある**	1	2	3	4	5
16^b^	**症状の軽減は、リカバリーの不可欠な要素である**	1	2	3	4	5
17^b^	**その人の病気や状態の深刻さに応じて、リカバリーへの期待や希望を調整する必要がある**	1	2	3	4	5
18^b^	**リカバリーの考え方がもっとも有用な人は、治療が一段落した人や治療をほぼ終えた人である**	1	2	3	4	5
19^b^	**治療に従えば従うほど、その人はリカバリーしやすい**	1	2	3	4	5
20	**重い精神の病気をもつ人や、アルコール·薬物乱用からリカバリーしつつある人も、精神保健の専門職者と同様に、他の人のリカバリーの手助けとなることができる**	1	2	3	4	5

^a^No. 1, 3, 4, 13の4項目は、16項目版日本語版尺度には含まれない。

^b^No. 2, 6, 7, 9, 10, 11, 14, 15, 16, 17, 18, 19の12項目は逆転項目として計算する。

## Abbreviations

RKI: Recovery Knowledge Inventory
RAQ: Recovery Attitudes Questionnaire
SDSJ: Japanese-language version of the Social Distance Scale
KMO: Kaiser–Mayer–Olkin
EFA: explanatory factor analysis
CFA: confirmatory factor analysis
GFI: goodness-of-fit index
AGFI: adjusted goodness-of-fit
CFI: comparative fit index
RMSEA: root mean square error of approximation
