# Correction to: The property of the Japanese version of the Recovery Knowledge Inventory (RKI) among mental health service providers: a cross sectional study

**DOI:** 10.1186/s13033-018-0214-2

**Published:** 2018-06-19

**Authors:** Rie Chiba, Maki Umeda, Kyohei Goto, Yuki Miyamoto, Sosei Yamaguchi, Norito Kawakami

**Affiliations:** 1 Research Institute of Nursing Care for People and Community, University of Hyogo, 13-71 Kitaoji-cho, Akashi, Hyogo 673-8588 Japan; 2 Department of Public Health Nursing, Graduate School of Nursing, St Luke’s International University, 10‑1, Akashi-cho, Chuo-ku, Tokyo, 104-0044 Japan; 3 Tokyo Musashino Hospital, 4-11-11, Komone, Itabashi-ku, Tokyo, 173-0037 Japan; 4 Department of Psychiatric Nursing, Graduate School of Medicine, The University of Tokyo, 7-3-1, Hongo, Bunkyo-ku, Tokyo, 113-0033 Japan; 5 Department of Psychiatric Rehabilitation, Institute of Mental Health, National Center of Neurology and Psychiatry, 4-1-1, Ogawahigashi-cho, Kodaira-shi, Tokyo, 187-8553 Japan; 6 Department of Mental Health, Graduate School of Medicine, The University of Tokyo, 7-3-1, Hongo, Bunkyo-ku, Tokyo, 113-0033 Japan

## Correction to: Int J Ment Health Syst (2017) 11:71 10.1186/s13033-017-0178-7

Following publication of the original article [1], the authors reported an error in note “a” of Table 5.

Originally, the incorrect table note was published as:

^a^ No. 1, 3, 4, 13の4項目は、16項目版日本語版尺度には含まれない

The correct table note is:

^a^ No. 1, 4, 5, 13の4項目は、16項目版日本語版尺度には含まれない

## References

[1] Chiba R, Umeda M, Goto K, Miyamoto Y, Yamaguchi S, Kawakami N (2017). The property of the Japanese version of the Recovery Knowledge Inventory (RKI) among mental health service providers: a cross sectional study. Int J Ment Health Syst..

